# Telemedicine, Telepsychiatry and COVID-19 Pandemic: Future Prospects for Global Health

**DOI:** 10.3390/healthcare10102085

**Published:** 2022-10-19

**Authors:** Susanna Marinelli, Giuseppe Basile, Simona Zaami

**Affiliations:** 1 School of Law, Università Politecnica delle Marche, 60121 Ancona, Italy; 2 IRCCS Orthopedic Institute Galeazzi, 20161 Milan, Italy; 3 Department of Anatomical, Histological, Forensic and Orthopedic Sciences, “Sapienza” University of Rome, 00161 Rome, Italy

**Keywords:** telemedicine, telepsychiatry, regulatory/legislative frameworks, ethics and medicolegal implications

## Abstract

There is no denying that demand for telemedicine and telepsychiatry services has been on the rise, as the COVID-19 pandemic engulfed the world and upset the daily lives and certainties of us all. Such growth, however, calls for a comprehensive analysis and assessment of the strengths and weaknesses inherent in such innovative approaches, which are bound to change and evolve as the fourth industrial revolution unfolds before our eyes. The authors have set out to analyze the complexities and distinctive features of telemedicine and telepsychiatry by focusing on the strengths and weaknesses of such approaches and analyzing research findings, recommendations, and guidelines by scientific societies and institutions, for the ultimate purpose of striking a tenable balance between technological innovations and the ethics and moral imperative of guaranteeing equal access to care for everyone, irrespective of social and financial status. The European regulatory and legislative scenario has been briefly outlined, and the standards for the medicolegal sustainability of such practices have been explored. Ultimately, in order to improve accessibility without compromising the quality of care, new broadly shared ethical standards, best practices, and guidelines need to be prioritized. National legislative initiatives and the international sharing of information need to be encouraged, for the ultimate purpose of optimizing and harmonizing telemedicine-based care for the sake of all patients. As technology moves forward and evolves, so must the normative standards and guidelines on which professionals must be able to rely when delivering telemedicine-based care in an ethically and legally viable fashion. From that perspective, addressing the digital divide means enabling more people to receive care and should therefore be seen as part and parcel of the effort to uphold the universal human right to health.

## 1. Introduction

The growing demand for telepsychiatry services during the COVID-19 pandemic is undeniable [1], but such growth requires a thorough analysis and assessment of the strengths and weaknesses inherent in such approaches, in order to fine-tune and optimize clinical practice by harnessing the benefits of the fourth industrial revolution unfolding before our eyes. Still, it is essential to bear in mind that only through evidence-based medicine rooted in standards and guidelines delineated by subject matter experts, can the integration of telemedicine in psychiatric care be fully implemented and thoroughly harnessed.

As the COVID-19 pandemic unfolded, telemedicine gradually became a considerably valuable service to avoid contagion between healthcare professionals and patients and has encompassed a growing number of medical disciplines [2], such as oncology and microbiology, in order to allow for the continuation of the study of the role of viruses in the etiopathogenesis of many diseases and forms of cancer [3], and for vulnerable patients such as those who have undergone kidney transplants [4].

In addition to fostering patient access to care, telemedicine is a powerful tool that enables patients and professionals to leverage technology and overcome the barriers which frequently get between patients and timely access to care. Many patients face socioeconomic barriers that decrease access to critical behavioral health services, e.g., they cannot gain access to transportation to get them to therapy sessions, they suffer mobility issues caused by comorbid conditions, or just lack the economic resources to regularly commute to their appointments [5]. Telepsychiatry can be effective for many people, providing an alternative option to traditional in-person psychiatric services [6]. That could prove even more beneficial in light of the fact that the COVID-19 pandemic has caused enormous mental and psychological suffering affecting millions of people all over the world, hence such new means are likely to be even more relevant and effective at catering to as many people in need of support as possible [7]. The authors have sought to succinctly analyze and expound upon the distinctive traits and complexities inherent in telemedicine and telepsychiatry. Such an analysis has been conducted by focusing on the strengths and weaknesses of such approaches and delving into research findings, recommendations, and guidelines by scientific societies and institutions. The ultimate purpose is striking a tenable balance between technological innovations and the ethical and moral imperative of guaranteeing equal access to care for everyone, irrespective of social and financial status, thus ensuring that the potential of telemedicine to expand access to quality care is available to as many people as possible worldwide. Although new technological innovations offer novel opportunities to widen our horizons, fast-moving technological advancements can in fact outpace our ethics frameworks and legislative/regulatory capabilities.

## 2. Materials and Methods

Relevant sources were identified in order to provide an overview of telemedicine peculiarities, distinctive functional traits, applications, and the current ethics/legal standards governing such practices, particularly in the European Union. Recommendations and guidelines by national and international scientific institutions have also been drawn upon, insofar as they addressed the policy-making, legal, regulatory and ethical implications, in addition to the delivery challenges. All sources exclusively focused on technical aspects and connotations have been excluded. A total of 65 sources have ultimately been deemed relevant, comprising research articles centered around telemedicine-related distinctive features and complexities (particularly within, but not limited to, the context of the COVID-19 pandemic) and recommendations, guidelines and directives by scientific societies and supranational institutions spanning the 2000–2022 time period. From such a body of research, the authors have set out to delineate the standards which ought to be met for the sake of the medicolegal tenability of telepsychiatry/telemedicine practices. Clean-cut criteria are in fact essential if we are to harness telehealth potential to its fullest while avoiding or limiting any form of discrimination in access and availability.

### 2.1. The Benefits of Telemedicine/Telepsychiatry Have Long Been Documented

Recent findings point to telemedicine as an option effective in enhancing client access to providers. The American Psychiatric Association [8] and the American Telemedicine Association (ATA) [9] have highlighted strong evidence suggesting how telepsychiatry can result in improved outcomes and high levels of patient satisfaction, also stressing that telepsychiatry matches in-person care in terms of diagnostic accuracy, therapeutic effectiveness, quality of care, patient privacy and confidentiality. In addition, telepsychiatry has been supported for the treatment of post-traumatic stress disorder and is indicated to be effective and acceptable in the treatment of depression, anxiety, eating disorders, substance abuse, and schizophrenia. Recent research findings have pointed to mobile apps and Short Message Service (SMS) and their advantages as therapeutic contributors for several mental conditions such as depression, anxiety, and stress [10], ultimately highlighting promising and emerging levels of efficacy in the use of telemental health applications and text messaging. On the other hand, the field is still evolving, and adaptation to each individual patient’s needs may not be as immediate, as evidenced by a recent study that concluded that some patients may not adapt as readily as others to the use of mobile apps [11]. A clinical update by the American Academy of Child and Adolescent Psychiatry (AACAP) has also remarked that preliminary surveys point out that home sessions based on teletherapy with youths and family members are likely to be well-suited and effective and could play an important role for young patients who cannot easily travel outside their homes or in order to guarantee continuity of care for families who relocate, e.g., military families [12].

### 2.2. Telemedicine within the Pandemic Context: Novel Challenges

Furthermore, research data have shown that as the COVID-19 pandemic has caused unprecedented strain on healthcare services almost all over the world, telepsychiatry, much like telemedicine in general, can undoubtedly constitute a remarkably valuable solution to the issues posed by physical distancing measures [13,14]. Such a potential can also substantially benefit patients with addiction and substance use disorders (SUDs) [15]. The COVID-19 pandemic has given rise to novel dynamics of drug abuse and addiction, with traditional trafficking avenues moving online, although such a shift was already underway before the pandemic broke out [16,17,18]. It could therefore be concluded that the criminal element itself has been taking advantage of remote interactions and transactions as the pandemic set in, exhibiting a considerable level of adaptability [19]. The rise in the consumption of novel psychoactive substances, extremely elusive and hard-to-detect substances intended to replicate the effects of illegal drugs of abuse [20,21,22,23] is worrisome and may pave the way for trafficking and addiction dynamics much harder to supervise and address over time with all the mental health implications such a shift could produce [24,25]. Telemedicine/telepsychiatry may be an extra arrow in our quiver to stem the tide of SUD pervasiveness as a major mental and public health threat and to reach many individuals in a state of vulnerability who would not otherwise seek care at health facilities. Nonetheless, although its use has substantially spread because of unique emergency circumstances, the move towards technology-mediated medical intervention had already long been established.

## 3. Results

### 3.1. European Union: Legal and Regulatory Inconsistency Can Put a Damper on Telehealth Options

Telemedicine is by its very nature a borderless concept. The potential of telemedicine as a cross-border avenue of care within the EU has been explored as a considerably promising approach enabling patients to be matched with the best-suited providers to meet their needs [26]. In the European Union, numerous directives have over the decades dealt with the rise of telemedicine services, among which it is worth mentioning the 2000 Electronic Commerce Directive [27], and the 2002 Directive regulating privacy enforcement and electronic communications [28]. Both are closely focused on information technology (IT) service provision, whereas health services, particularly inter-country, are covered prominently by the 2011 Cross-Border Directive [29].

The regulatory beacon, however, is represented by Guidelines 2/2019 on the processing of personal data under Article 6(1)(b) of the General Data Protection Regulation (GDPR) in the context of the provision of online services to data subjects [30], in keeping with Articles 56 and 57 of the Treaty on the Functioning of the European Union [31], which define and regulate the freedom to provide services within the European Union. Nonetheless, more adequately crafted, targeted, and comprehensive legal and regulatory frameworks will be needed in order to provide clinicians with solid standards of care in a field that evolves and outpaces regulatory and legislative interventions. To that end, telemedicine-related teaching should also be included in medical school curricula, so as to enhance the level of homogeneity and objectivity. Also, many telehealth incentives and funding put in place during the pandemic could be revoked. Legal complexities cannot be overlooked either: in spite of the numerous policy papers and documents released by European bodies and institutions over the years [32], an even and straightforward set of regulations aimed at governing telehealth in Europe is still missing [33]. That void, therefore, makes regulatory action towards telemedicine a prerogative of national governments and legislatures, although telemedicine is by definition without borders and EU patients could benefit from interventions from anywhere in the EU. After all, as it pertains to healthcare as a whole, such diversity is acknowledged by the Council of Europe itself in its Conclusions of 1–2 June 2006 on Common values and principles in European Union Health Systems [34]. According to that framework, there is a set of operating principles shared by health systems all across the European Union. Such operating principles are essential in order to build patient trust in cross-border healthcare, which in turn will foster patient mobility as well as ensure substantial degrees of health protection throughout the Union. That being said, the Council itself acknowledges in that very same statement that the practical ways and means by which such shared values and principles are turned into reality may vary significantly among the EU Member States. Hence, any regulatory and legislative framework defining the nature and amount of healthcare services to which citizens are entitled, as well as the mechanisms put in place to fund and deliver such services (e.g., the extent to which reliance on free market-based, competition-driven dynamics is acceptable to manage healthcare systems), must be taken into account within the context of national prerogatives and the broad margin of appreciation granted to member states.

### 3.2. The Issue of Governance

As a result, the governance of telemedicine has considerable variations among EU member states in terms of fields and the extent of application and regulation [35]. Such an uneven regulatory stance entails that some nations chose to apply to telemedicine a set of norms conceived for the realm of information technology (IT), whereas others passed pieces of legislation more befitting healthcare and related norms, or even according to social security statutes. It is therefore worth bearing in mind that the legal vacuum or legal inconsistency that still exists in Europe can detract from the effectiveness and applicability of telemedicine, by breeding uncertainty among patients [36] and healthcare professionals mistrustful of an ill-defined and poorly regulated healthcare option, involving jurisdiction-related issues, potential liability, the norms governing confidentiality and privacy of patient records [37,38]. Furthermore, for such new burgeoning dynamics to be truly beneficial, they should be coordinated with adequate funding/reimbursement frameworks and streamlined and targeted measures at the local and organizational levels, for the ultimate purpose of ensuring functional integration long after the pandemic is behind us. The risk that certain telemedicine technologies could be uneconomical or unaffordable to some patients who could otherwise benefit from such care modalities is real and needs addressing if we are to prevent inequalities in access from further exacerbating the health outcomes disparities between those with sufficient financial means and low-income patients.

### 3.3. Legal Governance Is One of the Still Lingering Barriers, According to WHO Analyses

The effort to meet the challenge of governing novel healthcare approaches based on innovative and fast-evolving technologies is not new. Such pivotal aspects were in fact brought to the forefront and stressed by the World Health Organization as early as 25 years ago, in a 1997 report by WHO Group Consultation on Health Telematics [39,40], which stated that “the delivery of healthcare services, where distance is a critical factor, by all healthcare professionals using information and communication technologies for the exchange of valid information for the diagnosis, treatment and prevention of disease and injuries, research and evaluation, and for the continuing education of healthcare providers, all in the interests of advancing the health of individuals and their communities”. At the same time, although the level of technology was very far 25 years ago from where it is nowadays, the WHO already saw the potential of telemedicine to give rise to legal and ethical challenges, recommending that “health telematics policies and initiatives take into account the relevant ethical and legal issues”. If such a caveat was relevant back then, it is all the more meaningful nowadays. To buttress those points, a recent and very thorough WHO analysis on telehealth feasibility has indicated legal issues as the number one barrier in the European Union, followed by cost, culture, and standards, whereas globally, the most prevalent barriers have been found to be costs, followed by legal, cultural, and infrastructure challenges [41]. In turn, such acknowledgments have culminated in the definition of the WHO Global Strategy on Digital Health 2020–2025, crafted to promote healthy lives and well-being for everyone, everywhere, at all ages. The fundamental purpose is therefore to harness the telehealth potential, through national or regional Digital Health initiatives guided by a soundly structured strategy integrating and fostering a synergy among financial, organizational, human, and technological resources [42].

## 4. Discussion

### 4.1. Telemedicine and Medicolegal Tenability

It would be remiss to overlook the peculiarities of telemedicine from a medicolegal perspective. In fact, as remarked by the World Medical Association (WMA) [43], telemedicine-based care must be grounded in solid fundamental principles governing all interactions between doctors, patients, and all stakeholders involved.

The informed consent process, for instance, ought to be molded so as to reflect the unique traits of telemedicine care in order to avoid “grey areas” that might not adequately serve the needs of patients and professionals and even result in litigation and malpractice allegations. In addition to explaining the medical interventions themselves, doctors are required to explain telemedicine modalities and ways of interaction [44,45]. That is maybe even more urgent when telepsychiatry is involved, given the state of emotional and mental fragility experienced by many psychiatric patients or their guardians. Patients will then have to be thoroughly informed as to the modalities by which telemedicine works, how consultations and appointments should be scheduled, all and any concerns relative to privacy safeguards, and the risk of technological malfunction possibly resulting in security breaches (with the potential loss or theft of personal data) [46,47].

### 4.2. Crafting Shared Protocols for Medicolegal Tenability

The elaboration of protocols for contact during telemedicine interactions will have to be fully clarified as well. In that regard, physicians or technical experts appointed by the legal entity in charge of telemedicine services are required to take all steps to preserve patient confidentiality, privacy, and data integrity. Unauthorized access and breaches of data and information produced during telemedicine procedures and counseling must be prevented by means of effective and up-to-date security measures in adherence to local legislation [48]. Electronic transmission of information must also be safeguarded against unauthorized access. Moreover, should the need arise for coordinating policies and care measures with other professionals, it ought to be conducted in a clear and straightforward fashion, without influencing the patient’s choices [49]. Certainly, the delivery of telemedicine services needs to be molded and fine-tuned according to local regulatory frameworks, which would likely require telemedicine platforms to be licensed in order to uphold patients’ best interests [50]. It is also of utmost importance to perform regular quality evaluations of all telemedicine procedures, which also should be tested in terms of their effectiveness, efficiency, safety, feasibility, and cost-effectiveness, in order to make sure that patient security and the best possible diagnostic and treatment practices are offered [51]. That assessment is even more important in emergency circumstances: strengths and weaknesses of telemedicine are in fact not yet fully clarified [52]. Regardless, should it be necessary to resort to telemedicine in an emergency scenario, factors such as the severity of the patient’s medical condition and the capabilities and competency of the persons who are with them obviously would play a role in the delivery of care, advice, and treatment interventions/recommendations [53]. Clarity is once again key: protocols for referrals to emergency services should therefore be devised and enforced by any entity legally in charge of implementing telemedicine services. A degree of objectivity arising from widely shared protocols, guidelines, and norms is key to ensuring that telepsychiatry, and indeed telemedicine as a whole, can be delivered in a tenable fashion from the medicolegal standpoint, which is the only way to uphold the patients’ rights and shield doctors and providers from malpractice allegations [54].

### 4.3. Telehealth as a Human Rights Tool

The relevance of such prescriptions is bound to grow further as telemedicine will come to rely on ever-more sophisticated and advanced technologies such as machine learning artificial intelligence and robotics [55]. By the same token, it is an ethical imperative to address the digital divide, which prevents access to telemedicine services for millions of people both in developing and wealthy countries. Benefitting from telemedicine in fact requires the reliance on internet access and the availability (and ability to operate) devices enabling patients to receive care. The technical and financial inability to utilize available technology and a lack of access to the internet can therefore constitute an element of discrimination that runs counter to the principle of universal access to healthcare and the inalienable human right to health as defined by the Office of the United Nations High Commissioner for Human Rights and the World Health Organization [56], and most prominently, by the 1966 International Covenant on Economic, Social, and Cultural Rights [57], i.e., the central instrument of protection for the right to health, which defines “the right of everyone to the enjoyment of the highest attainable standard of physical and mental health”. It is in fact no coincidence that in 2018 the United Nations Office on Drugs and Crime (UNODC) officially partnered up with blockchain-centered telemedicine and telepsychology company doc.com in an effort to expand basic telemedicine services across Eastern Africa [58]. A similar initiative has been recently launched by the United Nations Development Program (UNDP), which in 2021 launched “Doctor for Everyone”, a telemedicine tool based on a digital platform and relying on a smartphone application designed to foster access to medical examination and treatment at the grassroots level, and enable people, especially in mountainous, remote, and isolated areas, ethnic minorities, and people with disabilities to benefit from quality health services [59].

### 4.4. Core Message and Limitations

The present article has been conceived to shed a light on the importance of a legally and ethically tenable approach to telepsychiatry/telehealth and to raise awareness as to the urgency to achieve a degree of objectivity instrumental in optimizing the sound implementation of such techniques. If in fact healthcare professionals and services are often set back and hobbled by malpractice lawsuits of a frivolous nature, it reverberates on healthcare quality and the costs impacting all of the community, including low-income families and individuals. That is likely to be even more of a risk with telehealth services, due to the very peculiarities and applications involved. Hence, clean-cut ethical and legal standards in a field such as telemedicine are imperative, at least among countries that share a common set of core values, such as EU members. It is in fact worth bearing in mind that telehealth lends itself to transnational interventions that could make a difference for patients living in underserved countries or regions, for which barriers to care may penalize public health in a major way. As such techniques acquire new levels of sophistication, the opportunities and potential benefits are bound to grow, but so are the risks which may arise, for instance, from unorthodox interventions or unsafe personal data protection protocols. Ambiguity and hazy rules can therefore damage both patients and professionals/institutions, not to mention healthcare systems when public facilities are involved. If the opportunities and benefits of an increasingly borderless community of nations are to be harnessed to their full potential, patients and professionals will have to rely on frameworks aimed at delineating the exact scope, patterns, and limitations of telemedical interventions in terms of their implementation at all stages. For technological advancements to be ethically applied, in fact, it is of utmost importance that they are designed to reach and benefit as many people as possible, which is part and parcel of the very essence of all major international treaties and covenants in which the right to health is enshrined. Such a target is therefore non-negotiable according to the very tenets of medical practice. The study’s main limitation stems from its not including a compatibility analysis of national sets of legislation and regulations as it pertains to telehealth. Future research ought to be aimed at outlining the best avenues towards finding common ground among a given set of nations in order to optimize the criteria by which telemedicine services are delivered.

## 5. Conclusions

The pandemic has made it necessary to use telemedicine and telepsychiatry to meet the needs of patients who could not stop treatment during the emergency crisis period. In light of the fact that the pandemic has put an enormous mental and psychological strain on millions of people, such new means are all the more relevant to cater to as many people in need of support as possible [60].

Of fundamental importance are the training and clinical practice that jointly manage to support and implement the provision of telemedicine techniques [61] in order to guarantee an adequate capacity for patient involvement and constitute the bedrock of all healthcare interventions to meet the patients’ therapeutic needs. Despite the inconclusiveness of currently available data, telemedicine has proved to be an extremely valuable resource for guaranteeing continuity of care during emergency situations, effectively stimulating innovative research avenues to meet the challenges arising from ordinary care as well as emergency scenarios.

Hence, the implementation of remote consultation through telepsychiatry should be encouraged and technically improved by enrolling all stakeholders, policymakers, and industry officials. Particularly in pandemic times, telepsychiatry can provide an alternative to face-to-face assessment and can also be used creatively with other digital and in-person technologies such as the hybrid care model to create new avenues of care for patients with psychiatric disorders [62,63]. The aspects that still need improvement are common to telemedicine as a whole. Certainly, a risk exists that telemedicine may be considered an equal substitute for face-to-face healthcare services, and could even be incentivized mostly as a means to cut down on expenses and costs or as an ill-advised form of incentive enabling physicians to earn more through over-service, which may come to the detriment of patient care. In this regard, it will be necessary to improve safety with the use, training, and experience of digital devices in all modes for both doctors and service users [64,65], but also to raise awareness as to the true purposes and fundamental goals of telemedicine, i.e., to improve accessibility without compromising care. To that end, the development of broadly shared ethical standards, in the form of best practices and guidelines, national legislative initiatives, and the international sharing of information and data on all aspects and complexities related to telemedicine practices need to be encouraged, for the ultimate purpose of optimizing and harmonizing telemedicine-based care for the sake of all patients. Such concerted efforts are certainly in keeping with the principles and precepts enshrined in all major human rights treaties codifying the inalienable and universal right to health. As technology unremittingly moves forward and evolves (thus becoming instrumental to upholding the right to health), so must the normative standards and guidelines on which professionals must be able to rely when delivering telemedicine-based care in an ethically and legally viable fashion.

## Data Availability

Not applicable.
